# ST-CenterNet: Small Target Detection Algorithm with Adaptive Data Enhancement

**DOI:** 10.3390/e25030509

**Published:** 2023-03-16

**Authors:** Yujie Guo, Xu Lu

**Affiliations:** College of Computer Science, Guangdong Polytechnic Normal University, Guangzhou 510665, China; jennyguoyj@163.com

**Keywords:** small target detection, deep learning, selective oversampling, adaptive data enhancement

## Abstract

General target detection with deep learning has made tremendous strides in the past few years. However, small target detection sometimes is associated with insufficient sample size and difficulty in extracting complete feature information. For safety during autonomous driving, remote signs and pedestrians need to be detected from driving scenes photographed by car cameras. In the early period of a medical lesion, because of the small area of the lesion, target detection is of great significance to detect masses and tumors for accurate diagnosis and treatment. To deal with these problems, we propose a novel deep learning model, named CenterNet for small targets (ST-CenterNet). First of all, due to the lack of visual information on small targets in the dataset, we extracted less discriminative features. To overcome this shortcoming, the proposed selective small target replication algorithm (SSTRA) was used to realize increasing numbers of small targets by selectively oversampling them. In addition, the difficulty of extracting shallow semantic information for small targets results in incomplete target feature information. Consequently, we developed a target adaptation feature extraction module (TAFEM), which was used to conduct bottom-up and top-down bidirectional feature extraction by combining ResNet with the adaptive feature pyramid network (AFPN). The improved new network model, AFPN, was added to solve the problem of the original feature extraction module, which can only extract the last layer of the feature information. The experimental results demonstrate that the proposed method can accurately detect the small-scale image of distributed targets and simultaneously, at the pixel level, classify whether a subject is wearing a safety helmet. Compared with the detection effect of the original algorithm on the safety helmet wearing dataset (SHWD), we achieved mean average precision (mAP) of 89.06% and frames per second (FPS) of 28.96, an improvement of 18.08% mAP over the previous method.

## 1. Introduction

The current progression in the fields of deep learning (DL), image processing, and computer vision (CV) technologies has changed the thinking about different features of day-to-day living [1]. The DL method has given a strong foundation for target detection with consistent accuracy [2]. Target detection has become one of the most essential topics in the CV community, requiring object classification and localization [3]. It has a variety of applications including autonomous driving [4], medical lesion detection [5], intelligent security [6], disaster management [7], agriculture surveys [8], urban planning [9], geographic information system updating [10], and many more. Small target detection task scenarios have important application value in various fields. In the field of manufacturing, target detection algorithms are needed to help people find defects [11] and deformation of small parts in the process of part assembly. In intelligent security, it is frequently necessary to detect remotely whether workers are wearing safely helmets correctly, while images of scattered workers present only dozens of pixels, or even fewer pixels. With these difficulties, small target detection demands to be solved for intelligent security.

However, detecting small targets is still a challenging problem. There is still a large performance gap between small and normal scale targets. Taking RetinaNet [12], one of the state-of the-art (SOTA) target detectors, as an example, it achieves 44.10 and 51.20 mAP on targets with medium and large sizes but only obtains 24.10 mAP on small targets on COCO [13] test-dev set. Such degradation is mainly caused by three factors: (1) The inherent scale of the target to be detected in some tasks is small [14], and the detailed information is difficult to find [15]. (2) The long shooting distance [16] leads to the small scale of the target to be detected, and the target occlusion or truncation caused by the movement of the device and jitter [17], the change of the shooting perspective [18], and the different brightness of the screen [19] also further increase the difficulty of small target detection. (3) The receptive field on low-resolution features may not match the size of small targets as pointed out in [20].

The definition of “small target” varies in different scenarios and there is not yet a unified standard. The existing small target definition methods are mainly divided into the two categories of relative scale and absolute scale. Relative scale refers to the definition of small targets from the perspective of the relative proportion of the targets and images [21]. However, these definitions based on relative scale have many drawbacks, which are affected by data preprocessing and model structure, and cannot effectively evaluate the detection performance of models for objects at different scales. The absolute scale refers to the definition of small objects in terms of their absolute pixel size. Since the selected data set only selects the head part of the target person for the box selection of the target box, it contains less information. Considering this factor, we adopt the definition based on absolute scale to determine the small target selection criterion, and define the image less than 32 × 32 as a small target [22].

CenterNet [23] recently has been reported as achieving SOTA performance in classification. Therefore, it is natural to attempt to extend its usage to detection tasks. CenterNet, with a simplified structure and strong expansibility, has a definite impact on the above problems. However, CenterNet has some flaws, such as predicting only through the last feature layer, resulting in the loss of target feature information and ineffectiveness in detecting small targets.

To address the above problems, we present a novel small target detection network with adaptive data enhancement (ST-CenterNet) for accurately increasing target samples directly and enhancing shallow semantic information. First, we design a novel selective small target replication algorithm (SSTRA) method to scale limited small targets. Then, shallow semantic information enhancement is performed by our proposed target adaptation feature extraction module (TAFEM), and this TAFEM uses the residual network ResNet and AFPN successively connect to retain more shallow features. Our main contributions of this paper include:

1. We propose a SSTRA algorithm to select all small targets by pixel filtering. By copying the identified small targets, it can effectively increase the number of small target samples.
2. We propose TAFEM to obtain feature maps of multiple receptive fields [24], by combining ResNet [25] with the adaptive feature pyramid network (AFPN) to retain more complete semantic information of shallow features.
3. A novel structure, AFPN, is proposed to enhance feature extraction and fusion, allowing TAFEM to detect small target regions more accurately.

The rest of this paper is arranged as follows: Section 2 covers the overview of the two main categories of current algorithms for deep learning-based target detection, and then introduces several main improvement directions of the small target detection based on deep learning. Section 3 is dedicated to the dataset and its details. The structure of the ST-CenterNet network described in the study is shown in Section 4, followed by a description of the network’s strategies. Section 5 explains the basic configuration in the experimental environment. Section 6 focuses on the analysis and results of the ablation experiments and comparison experiments using the ST-CenterNet network. The work results are discussed in Section 7, and conclusions are presented in Section 8.

## 2. Related Work

With the massive progress of convolutional neural networks (CNN), it has become mainstream in target detectors to adopt the modernized state-of-the-art CNN models as feature extractors [26]. The target detection methods are mainly divided into two-stage detection and one-stage detection algorithms. Two-stage detection algorithms, such as Faster R-CNN [27], Mask R-CNN [28], region-based fully convolutional networks (R-FCN) [29], and AAAI [30], use strategies such as selective search (SS) [31] to generate proposals that may contain targets of interest, and then match proposal features with the template. The matched proposals are obtained through classification and regression of proposal regions [32] and treated as the final target positions. One-stage detection algorithms (for example, single shot multibox detector (SSD) [33,34], YOLO series [35,36], RetinaNet, CornerNet [37], CenterNet), can directly obtain target position and category information by inputting images into the convolutional neural network.

Large numbers of detectors in deep learning have been designed for general targets. However, the insufficiency of small target detection and the extraction of feature information based on deep learning lead to unsatisfactory effectiveness. Small target detectability remains to be improved.

Data enhancement methods enhance the value of limited data. Mishra et al. [38] used the method of data transformation to increase the sampling rate of samples, but it may select poor samples and increase its detection error rate. Zhang et al. [39] added two sample-label data pairs proportionally, and generated new sample-label data. Chen et al. [40] proposed a dynamic scale training paradigm, which can dynamically guide data preparation through information feedback from the optimization process, and alleviate the challenge of scale transformation in object detection.

There have been some methods that have achieved some effects through feature enhancement. SSD detects targets with different scales and aspect ratios utilizing a multi-scale feature pyramid. The method uses convolution layers to predict the category fraction and the position offset of bounding boxes. Then, it obtains positioning bounding boxes by a non-maximum suppression post-processing strategy [41]. The module improves accuracy and efficiency to a certain degree but has poor detection performance for small targets. Therefore, improvements have been suggested to enhance the detection performance of SSD for small targets. The improved feature fusion single shot multibox detector (FSSD) [42], introduced by Li et al., added a light-weight feature fusion module based on SSD to generate a new feature pyramid detection model. Subsequently, the SSD was extended to the deconvolutional single shot detector (DSSD) [43], which up-samples low-resolution features, mainly through transposed deconvolution in the decoder [44].

Nonetheless, the deep convolutional networks are prone to lose the key position information of small targets when extracting feature information. Some algorithms determine the location region by the contextual information [45] of targets [46]. Lin et al. [47] introduced a multi-scale fusion strategy of a feature pyramid network (FPN) [48] to extract and fuse features at different scales, and obtained deep semantic information and shallow position information. Chen et al. [49] considered different feature extraction methods based on depth and shallow features to improve the detection effect of small targets. Ma et al. [50] proposed a new model that focuses on learning the deep features produced in the latter part of the network and made full use of the semantic and contextual information expressed by deep features. Huang et al. [51] presented a cross-scale feature fusion method that achieves an enhancement of contextual connection by using cross-scale feature mappings. However, direct cross-layer fusion may lead to a position offset and aliasing effect.

Aiming at these problems, some algorithms have proposed an attention mechanism [52] to improve feature information extraction. Attentional feature fusion (AFF) [53] adds local channel contextual information to the global channel through attentional feature fusion [54], to overcome semantic differences and scale inconsistencies among inputting features. Yu et al. [55] adopted dense connected convolutional networks to improve the ability of feature information extraction and to further enhance the contextual semantic information in shallow features. RFBNet [56] constructed receptive fields and a multi-branch convolution layer. The method used dilated convolution to expand the receptive fields of feature mappings, which improved the detectability of small targets to a certain extent.

Some algorithms replace the sparse coding labels with dense coding labels based on angle classification [57,58]. The arbitrary rotating rectangle is approximated into a two-dimensional Gaussian distribution [59]. Isokinetic rotation features were extracted by combining the isokinetic rotation network with detectors, aiming to solve the problem of multi-angle detection caused by the complex position information of small targets [60]. While these algorithms improve the performance of small target detection to some extent, they do not focus attention on increasing the number of small targets and simultaneously improving the capability to extract feature information. Related improvement methods are summarized in Table 1.

CenterNet is a one-stage target detection model with high accuracy. Target features are extracted from the inputting images by the baseline network and then introduced into the fully convolutional networks (FCN) to obtain a heatmap. Peak points of heatmaps are the centroids of targets. The position and category attributes of targets are obtained by using centroid location regression, which transforms the target detection problem into a key point prediction problem.

Motivated by these studies, we propose the ST-CenterNet model to expand small target samples based on the original dataset and to strengthen the extraction of small target feature information, improving the capability of small target detection.

## 3. Safety Helmet Wearing Dataset

### 3.1. Dataset Construction

Experiments are carried out on the open-source safety helmet wearing dataset (SHWD), which is used to provide head detection and safety helmet wearing. Referring to the standards of the PASCAL VOC dataset, an image dataset containing multiple scenes and targets was established, so that the target detection model has detection ability in different scenes, so as to facilitate the model for training, testing and analysis. The training and testing dataset contains 7581 images in which the targets are divided into two categories: hat and person. Among them, including 9044 people wearing helmets correctly (correctly worn, hat) and 111,514 people without helmets (not worn, person).

The images were acquired under complex lighting conditions, and there are complex backgrounds and small targets. Unlike the targets in natural scenes which are often taken from horizontal perspectives, images of the building site in SHWD are typically taken from several positions in diverse directions. The adopted dataset is inundated with low-resolution images with significant amounts of distractors and confusing target orientation. Moreover, our labeled frame only shows the head part of the workers, which further makes it difficult to detect whether a helmet is worn or not. These difficulties suggest that targets in SHWD usually are of diverse sizes and orientations with complex backgrounds, making them an excellent dataset for detection in complicated scenes.

The images in the dataset contain various angles, multiple and single targets, distances, occlusions, etc. The used public dataset is an xml file in PASCAL VOC format and was converted into a txt tag file in the CenterNet format. The dataset was labeled with labelImg for image targets, and the entire dataset was randomly divided into a training set and a test set, with the ratio of 8:2. The number of training set images in the 7581 images dataset is 6064, and the number of test set images is 1517.

### 3.2. Dataset Processing

The performance of detection results for small targets is weak due to the insufficient number of small targets in SHWD. Therefore, the network model will pay more attention to the training of medium and large targets during training, and ignore small targets, resulting in unsatisfactory detection effect of small targets.

**Figure 1:**
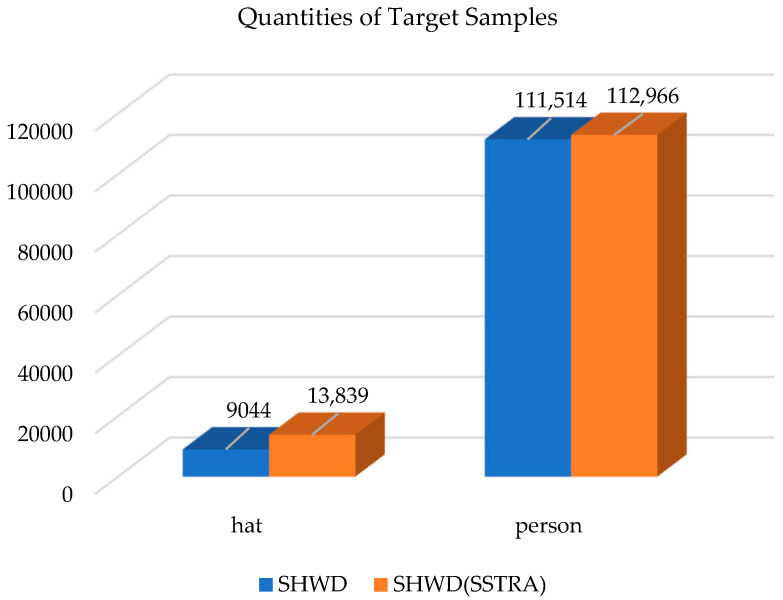
*The quantities of target samples on SHWD and SHWD processed by SSTRA.* In the legend, the histogram represents the proposed enhancement of SSTRA on SHWD. The figure demonstrates the quantities based on different categories between SHWD (the blue rectangle) and SHWD processed by SSTRA (the orange rectangle).

In order to enrich the detection background and increase the number of small targets in SHWD, the proposed SSTRA is used to replicate and flip the small targets in the dataset preprocessing stage. Targets with image resolution less than or equal to 32 × 32 are considered small targets. Figure 1 demonstrates the comparison results of differences between the number of targets without SSTRA processing and the number of targets with SSTRA processing in the dataset. The blue rectangle shows that the SHWD dataset has included 9044 targets into hat, 11,514 targets into person. The orange rectangle shows that the SHWD processed by SSTRA has included 13,839 targets into hat, 112,966 targets into person. It can be found that adding the SSTRA selected processing function effectively optimized the model performance and increased the numbers of hat and person.

**Figure 2:**
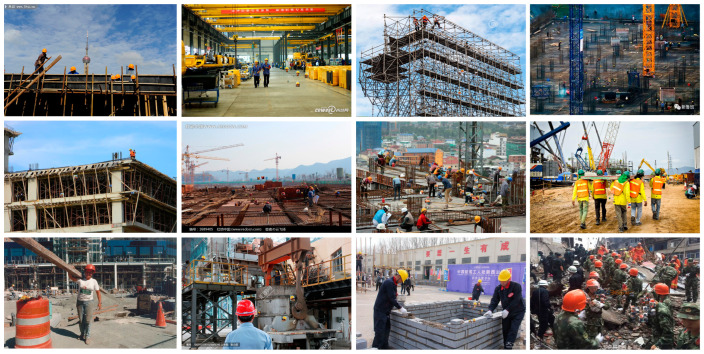
*Partial image processed by selective small target replication algorithm.* The process involves adding the number of small targets by replication and horizontal flipping for training target detection models. We apply random position replication to all training images with small targets, then replicate and flip all small targets in the image and put them back in the original image according to the initial proportion, without blocking the original small target.

To further verify the effects of SSTRA, we represent the visual results of images processed by SSTRA in Figure 2. There are a total of two targets in the first test image in SHWD, including two positive samples (hat). SSTRA achieves the purpose of enriching training samples by resampling and flipping small targets. After the SSTRA processing mechanism, there are a total of six samples in the first image, including six positive samples (hat). It can be seen from the above that, the SSTRA processing mechanism can effectively increase the number of samples in the SHWD dataset.

In the experiment, our SSTRA detects and replicates only the header information of objects, because the specific category can be judged only through the detection of header information in the experimental detection stage. Our proposed SSTRA is implemented through the real box annotated by the dataset, then by finding the small target according to the size of the targets, and finally by copying the bounding box of the small target. Since the dataset is labeled with the header, subsequent operations are processed against the target header.

## 4. ST-CenterNet

### 4.1. Network Structure

Our proposed ST-CenterNet algorithm samples an inputting image of uniform size by the feature extraction network. The feature information extraction network subsequently extracts the target feature information. After that, the extracted information is passed into FCN. The target centroid, the width and height of targets, and the offset value of the centroid for heatmap prediction are obtained. The position, size and category of targets are obtained by centroid regression.

The SSTRA is proposed to address the shortage of the number of small target samples in the dataset. Targets with pixel values less than or equal to the number in the images are selected and considered as small targets. Then, all small targets are replicated and flipped to achieve the oversampling of small targets, thus increasing the sample size for them.

Utilization of low-level features is one way to pick up information about small targets. The backbone network uses ResNet-50 to down-sample the image and combines it with AFPN to obtain feature maps of different sizes. This allows more complete extraction of feature maps with strong deep semantic information and shallow position information during inference processing.

**Figure 3:**
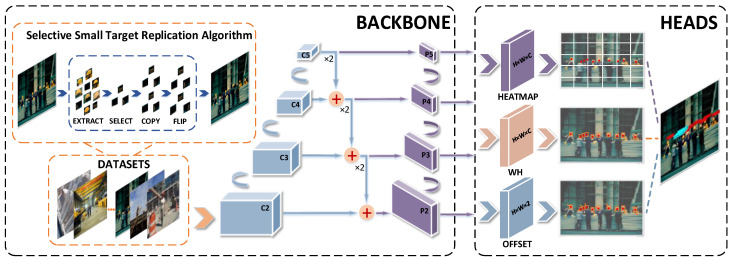
*The network structure of ST-CenterNet.* The dotted box on the left shows the execution process of the feature extraction network, in which the orange dotted box represents the processing method of the SSTRA (see Figure 4). After the feature extraction network is extracted, the feature is transmitted to the dotted box on the right for prediction (see Figure 5). The prediction is divided into three parts, which are the heatmap prediction, the width-height prediction, and the center point offset prediction.

Compared with the conventional size targets, small targets have fewer available pixels, which makes it difficult to extract complete feature information. With the increase of network layers, their semantic information and position information are gradually lost and become difficult for the network to detect. The features cannot provide effective expressions of semantic information. To deal with that, multi-scale learning is used, as a strategy to effectively integrate two types of feature information and perform better semantic representation. According to [43,47], it is appropriate to use separate groups of features to model distinct factors. One concern is that the shallow feature information required by small target detection can be easily diluted in the extraction process. To prevent this, we propose the TAFEM. The combination of ResNet-50 and AFPN is adopted to further enhance the performance of extracting deep semantic feature information. The enhanced feature information allows a better fusion of deep and shallow feature information to obtain more complete small target feature information. Subsequently, the outputting fusion feature is used for prediction, which is divided into three parts for generating a heatmap of key points, scale prediction of the bounding box, and offset of central points. Finally, the position of the target is estimated by the predicted center point coordinates. The network structure diagram of ST-CenterNet is shown in Figure 3.

In the proposed algorithm, the loss function is divided into the following three parts: the loss of the heatmap, the loss of the width and height of the bounding box, and the offset loss of the central key point. The loss function is formulated as follows:

(1) $$L_{SHL}=L_{H}+\lambda_{SIZE}L_{SIZE}+\lambda_{OFF}L_{OFF}$$

The notations $L_{H}$, $L_{WH}$, $L_{OFF}$ denote the loss value of the heatmap, the loss value of the width and height of the bounding boxes, and the offset loss value of the center point, respectively. We set $\lambda_{WH}$ as 0.1 and set $\lambda_{OFF}$ as 1 unless specified otherwise.

When calculating the loss value of the heatmap, the idea of focal loss [37] is used for reference. Focal loss is mainly to solve the problem of imbalanced classification in target detection. For the samples that may complete the classification, we take appropriate measures to reduce the proportion of training. We use L1 loss at the length and the width of bounding boxes. Spatial resolution of the feature map output by the backbone network becomes one-quarter of the original inputting image. It is equivalent to a pixel in the outputting feature image corresponding to the 4 × 4 region of the original image. Therefore, it is essential to introduce offset loss. We adopt the L1 loss function to predict the centroid offset loss for an improvement of the accuracy of centroid coordinate prediction.

### 4.2. Selective Small Target Replication Algorithm

The number of small target samples in SHWD is less than that of general targets. This leads us to pay more attention to the training of large and medium-sized targets, neglecting the training of small targets when using the network model for training. Because of the small size of small targets or the small proportion in the image, the position diversity of small targets in the image is insufficient.

**Figure 4:**
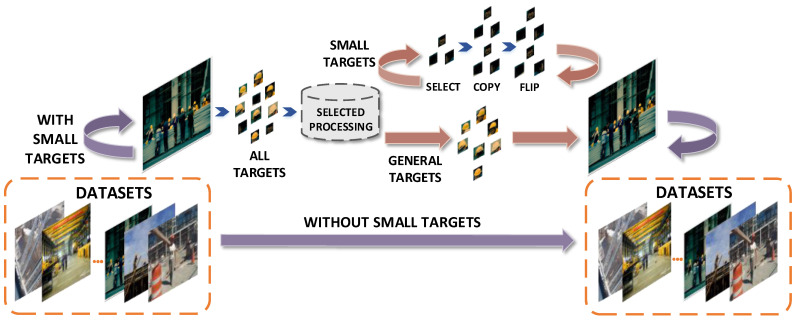
*Illustration of the proposed SSTRA.* The algorithm calculates the size of all targets and then screens the pixel value. Selected processing mainly solves replication and horizontal flipping based on the boxes of small targets. Using the above operations, we expect to directly increase the sample size of small targets through oversampling.

Considering the above two problems, we utilize SSTRA to selectively oversample all targets in the image and to achieve separate replication of small targets to enrich their sample size. Extracted targets are used to obtain targets with pixel values less than or equal to 32 × 32 against the background of a construction site by screening. These are considered small targets. All of the original images that contain small targets are determined and then individually extracted. The extracted small targets are replicated twice and horizontally flipped at the second copy, and then are put back into the corresponding original images according to initial sizes of extraction. Related information about these small targets is added to the extensible markup language file corresponding to the image. The architecture of SSTRA is demonstrated in Figure 4.

Our SSTRA focuses on two replications of small targets, which individually match pixel value screening. Then, there is repeated sampling processing of small targets to obtain a larger number of samples and to further increase the diversity of positions to improve the detection performance of small targets.

### 4.3. Target Adaptation Feature Extraction Module

**Figure 5:**
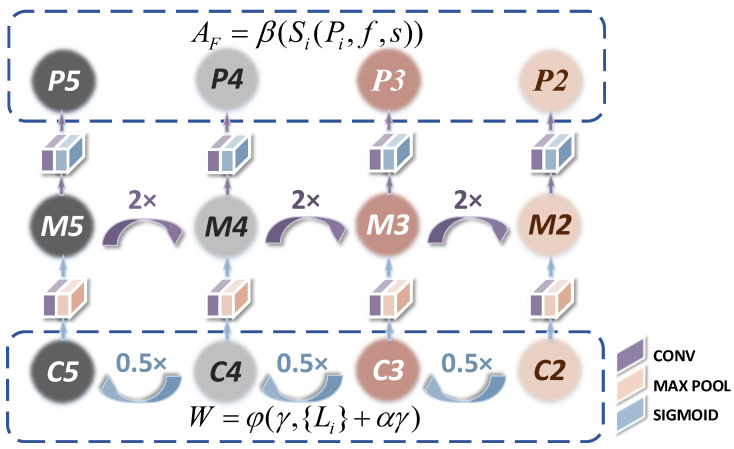
*The structure of AFPN.* To perform down-sampling, we use ResNet-50 to extract residual mapping and identity mapping ${(C}_{2}{,C}_{3}{,C}_{4}{,C}_{5})$ at each layer. We up-sample with AFPN and the deep features ${(P}_{5}{,P}_{4})$ and shallow features ${(P}_{3}{,P}_{2})$ are obtained by fusion of the horizontal connection.

The detection task requires models to identify targets on more scales than the classification task requires, in order to preserve information from various layers. This is especially important for detection because each layer has different receptive fields. CenterNet [23] makes predictions based only on the last feature layer, which disregards details of the shallow features, resulting in poor performance in detecting small targets. As shown in Figure 5, our TAFEM uses ResNet-50 to down-sample images. The performance of the detector is degraded because of couplings of features at different scales and the mismatch between pyramidal layers and target sizes. The AFPN mainly adopts decoupling of detection of different sizes. In particular, it can be used for small targets with high-resolution feature mapping; it allocates more appropriate feature levels for them. It assigns small targets to the corresponding layer to obtain more comprehensive region information.

The AFPN constructs a four-layer feature pyramid by up-sampling the deep convolution feature map and fusing this with the shallow features through horizontal connection. The details of TAFEM are shown below.

For the network structure of the deeper layers, the parameters are generally set close to zero during initialization. This leads to problems such as gradient explosion as the network layer deepens during training to update the parameters of the shallow-layer network. The result may make it impossible to update the parameters of the shallow-level network. We set up a linear combination of the network layers based on the original network structure. With an increased number of network layers, the linear combination for the mapping is as follows:

(2) $$W=\varphi \left(\gamma ,\left\{L_{i}\right\}\right)+\alpha \gamma$$

The notation W denotes the output vector processed by linear combination of feature layers. The notation $\varphi \left(\gamma ,\left\{L_{i}\right\}\right)$ denotes multiple convolutional layers, where φ is the sigmoid function, γ is the input vector of the feature layer, and $L_{i}$ is the weight layer. When both φ and γ have the same dimension, we perform an element-wise addition of the inputs and outputs between layers. The square matrix α is used for concrete linear operations. We set α as 1. However, when they have a different dimension, we perform a linear projection before addition.

The feature information of each predicted feature map is enhanced by boosting each feature map to the same number of channels by convolution with a convolution kernel size of 1. It is then fused backward and forward with the shallow features by lateral connections, which are similar to the jump connections in the residual structure. The features of each layer obtained after independent up and down-sampling are passed to an activation gate consisting of a combination of multiple convolutions and sigmoid activation functions. Thus, generated lateral features are used to fuse location features with semantic feature information:

(3) $$A_{F}=\beta \left(S_{i}\left(P_{i},f,s\right)\right)$$

where β denotes the sigmoid function, $S_{i}$ denotes the convolution corresponding to the $i^{th}$ layer, $P_{i}$ denotes the characteristic layer obtained by sampling corresponding to the $i^{th}$ layer, and ${s=2}^{i-1}$ denotes the convolution stride.

Deep and adjacent feature layers are added and element-wise multiplication fusion processing is conducted. Convolution is used to eliminate the aliasing effect of the up-sampling and to generate a new feature map according to the sizes of the targets:

(4) $$P_{i+1}=P_{i}+\lfloor log_{2}\sqrt{\frac{wh}{512}}\rfloor$$

The notations $P_{i}$ represent each pyramid feature layer corresponding to the $i^{th}$ layer; w and h are the width and the height of the bounding box of targets, respectively; and 512 represents the uniform size of the input image.

After the convolutional layers, the three feature maps (in Figure 3) are flattened into vectors and then concatenated together for recognition. By combining low-level, high-resolution information with high-level, strongly semantic information, AFPN allocates feature maps of different levels according to the targets at different sizes. It adapts to various receptive fields and improves the feature extraction ability of targets.

## 5. Experimental Settings

### 5.1. Experimental Configuration

In order to conduct model training and performance testing, we set up the experimental environment configuration. Detailed experimental settings are presented in Table 2.

### 5.2. Parameter Settings

We adopt a stochastic gradient descent (SGD) algorithm for model training to update and optimize the weight of the network model. The model training uses synchronous batch normalization [61]. According to the training parameters with good models and the memory capacity possessed, the number of iterations is set to 200. Model parameters are set to batch size of 10, weight decay of 5 × 10^−4^, momentum factor of 0.9, and a step learning rate decay [62]. In the training of the CONVOLUTIONAL neural network, the learning rate is set to be constant at 1 × 10^−4^. The updated formula of the weighting is as follows:

(5) $$W_{i+1}=W_{i}-\varepsilon *\eta$$

The notations $W_{i}$, $W_{i+1}$ denote the immediate parameter, and the updated parameter, respectively. The notation ε is influenced by an intermittently increasing parameter, which indicates the number of times that the performance of the tolerant network is without improvement. We set the intermittently increasing parameter as 2, and take the learning decay rate ε as 0.5. η denotes the learning rate.

To speed up the training, the backbone of the proposed model is initialized from a checkpoint pre-trained by ResNet-50 with self-training [63]. All other results are from models with random initialization, unless otherwise stated. We carry out sample shuffling and divide the dataset into 10 parts. The images are assigned to the training set and verification set according to the ratio of 8:2. During the training, the backbone network ResNet-50 that we used has pre-training weights. Some of the pre-training weights applied to the network are generic. It should be stressed that the backbone network is frozen first and more resources are placed in the network parameters in the later part of the training. This makes the training procedure more convenient to apply and brings better performance over time and resource utilization. After a period of network parameter training, the frozen part is thawed and all are trained together. In the frozen stage, we freeze the main stem of the model. The feature extraction network does not change, resulting in a small amount of video memory being occupied. The network is just fine-tuned at this time. In the unfrozen stage, the backbone of the model is not frozen. This changes the feature extraction network and takes up more video memory, resulting in a changeableness of parameters in the network. We take the iteration number of the frozen part as 100 times. Similarly, the iteration number of the unfrozen part is 100 times.

### 5.3. Evaluation Metrics

According to the standard protocol defined by the public dataset, we adopt the mean average precision (mAP), precision, recall, and f1-score to evaluate the detection accuracy of the algorithm in SHWD. We use frames per second (FPS) as the evaluation index of detection over speed. Because we expect to detect whether the remotely operating workers are wearing their helmets correctly, we also use the evaluation metric in pedestrian detection, log-average miss rate (MR^−2^), to evaluate the performance of the proposed detector.

## 6. Experimental Results

### 6.1. Comparative Experiment and Analysis of Results

**Figure 6:**
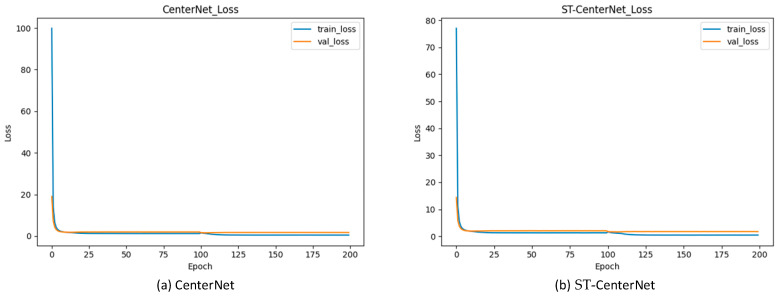
*Comparison of the loss function.* The desired result is a convergence in the value of loss function when training and validating the model. Figure demonstrates the training and validation loss function values between CenterNet (**a**) and our method ST-CenterNet (**b**).

When training and verifying the ST-CenterNet model, the change curve of the loss function was drawn through the training and verifying results information of each round, as shown in Figure 6. The change of the loss function of the CenterNet model is represented by the left bar. The change of the loss function of the ST-CenterNet model is represented by the right bar. It can be seen from Figure 6 that the initial loss value of the ST-CenterNet model was smaller than the initial loss value of the CenterNet model.

Most images in the dataset are small targets with disordered directions, complex backgrounds, and different scales. Specifically, Table 3 compares the detection performance of various target detection algorithms with the proposed algorithm on SHWD. The same experimental environment and dataset were used for comparative experiments. The experimental data of the algorithm in the following table are based on the enhancement of the algorithm using SSTRA. The experimental results on the safety helmet wearing dataset show that the proposed method has reasonable detection results compared with other state-of-the-art methods. As can be seen in Table 3, the highest detection accuracy of CenterNet algorithm is 70.98%, and the highest detection accuracy of the proposed ST-CenterNet is 89.06%, which is an improvement of 18.08% in detection accuracy.

The experimental results of ST-CenterNet show that adding data enhancement and feature enhancement modules can improve the accuracy of CenterNet’s target detection. However, this will inevitably increase the amount of calculation and model parameters, resulting in a decrease in the detection speed.

**Figure 7:**
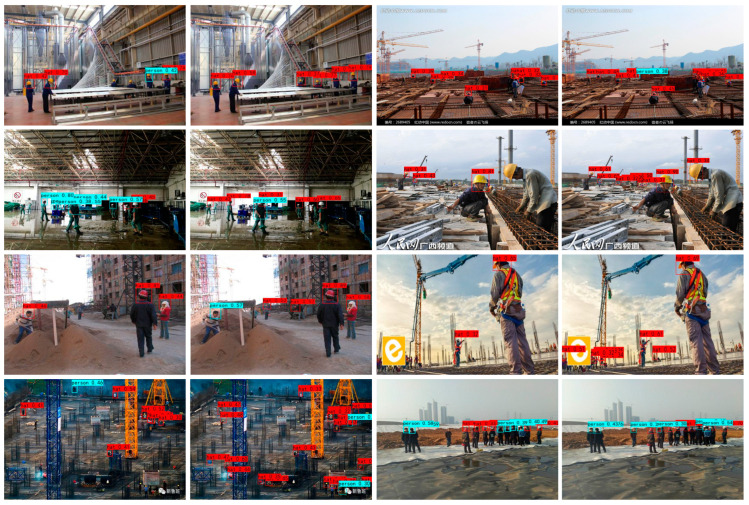
*Visualized comparison of the proposed algorithm with the original algorithm on SHWD.* We use pairwise comparison to demonstrate the effectiveness of the proposed algorithm for small target detection, in which the left image in each pair denotes the detection results of the original algorithm, while the image on the right -denotes the detection results of the proposed algorithm The figures with red boxes are the confidence of the detection result for correctly wearing the safety helmet, and the figures with blue boxes are the confidence of the detection result for not correctly wearing the safety helmet.

To more intuitively verify the target detection effect and model robustness of the proposed ST-CenterNet model, we present the visual results of CenterNet and ST-CenterNet for pairwise comparison in Figure 7. As shown in Figure 7, this demonstrates the comparison of the qualitative detection effect between the original algorithm and the proposed algorithm on SHWD. The selected images (in Figure 7) are the portion of the dataset that contains small targets that are difficult to detect. The left side of the two contrasted images shows the detection results obtained by the original algorithm, and the right side shows the detection results obtained by the proposed algorithm.

There are a total of six targets in the first test image, including six positive samples (hat). The CenterNet model detected a total of four targets, two missed detections, and one false detection. The ST-CenterNet model correctly detected a total of six targets, zero missed detections, and zero false detection. It can be seen from the above that, in the CenterNet model, there were serious false detection and missed detections for small targets and occluded targets. Compared with the original model, the qualitative detection results (in Figure 7) illustrate that, compared with CenterNet, visual results obtain a similar but significantly better performance for both SSTRA and TAFEM. The small targets that cause disorientation and are difficult to detect can be detected more accurately, which further proves that the adopted method effectively improves the detection performance of the model for them.

### 6.2. Ablation Experiment and Analysis of Results

According to the different improvement schemes, we conducted ablation experiments to explore the effects of the SSTRA module and TAFEM module on the model performance. The results of the ablation experiments are shown in Table 4.

In Table 4, the first line shows that CenterNet, the original algorithm, uses the original dataset. The second line ‘CenterNet+SSTRA’ illustrates the data enhancement using our SSTRA based on the original algorithm in the first line ‘CenterNet’. In the third line ‘CenterNet+TAFEM’, the feature extraction enhancement module TAFEM is added based on the original algorithm in the first line. In the fourth line ‘ST-CenterNet’, the TAFEM module is used for feature enhancement while SSTRA is used for data enhancement. Therefore, it can be seen from the table that SSTRA and TAFEM can each improve certain aspects of detection performance, and the combined effectiveness of SSTRA and TAFEM has more significant detection performance.

As can be seen from Table 4, the detection effect is 13.90% better than the original method on mAP. This indicates that the method of increasing the number of small targets, which replicates small targets, improves the target detection results. The TAFEM module makes deep and shallow features fusion, which introduces more accurate positioning information for each prediction layer. Compared with the original algorithm, the detection effect on mAP is improved by 13.22%, which proves that the proposed module can effectively fuse shallow details and deep semantic information. The performance of contrast images enhances the semantic information and feature expression ability of shallow feature images and improves the result of target detection. AFPN can focus on targets with specific sizes rather than wide backgrounds, effectively improving the detection performance. By adding the two proposed modules simultaneously, the detection effect of the proposed algorithm obtains a significant enhancement of 18.08% on mAP, compared to that of the original algorithm.

**Figure 8:**
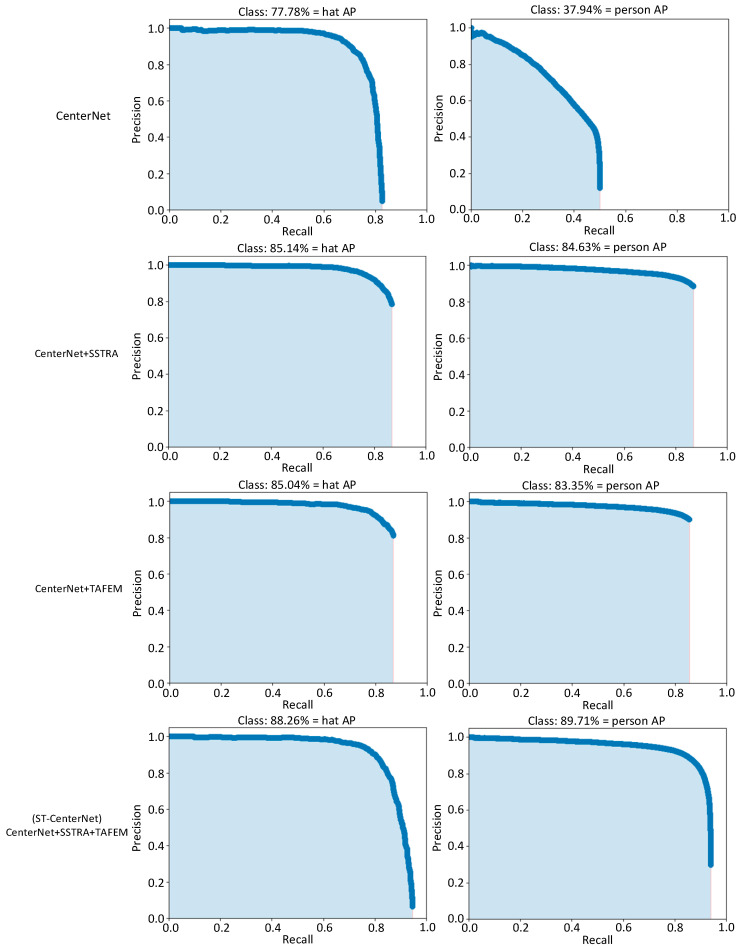
*AP curves for hat and person.* The figure demonstrated the effectiveness of each module in the ST-CenterNet model on the detection accuracy. The desired result is to increase the area occupied under the Precision-Recall curve for each target class (hat and person).

Four groups of models were trained, respectively, and each module was successively added on the basis of the CenterNet model. Four groups of models were tested in turn on the same test set, and the AP curves of hat and person detection are shown in Figure 8.

Compared with the results of “CenterNet”, the AP values of the “CenterNet+SSTRA” and “CenterNet+TAFEM” model were slightly improved. It was found that adding the SSTRA selected processing function effectively optimized the model performance and improved the detection accuracy of the model by increasing the number of small targets. The extraction of effective feature information and multi-scale feature fusion enabled the model to better detect targets. It can be found that adding the TAFEM feature enhancement function optimized the feature extraction efficiency and improved the detection performance. Compared with the “CenterNet+SSTRA” model, the AP of the “ST-CenterNet” was increased by 3.12% and 5.08%, respectively. Compared with the “CenterNet+TAFEM” model, the AP of the latter was increased by 3.22% and 6.36%, respectively. On the whole, each module in the proposed ST-CenterNet model effectively improved the detection accuracy, met the actual detection requirements, and verified the feasibility of the model.

### 6.3. Performance Validation

**Figure 9:**
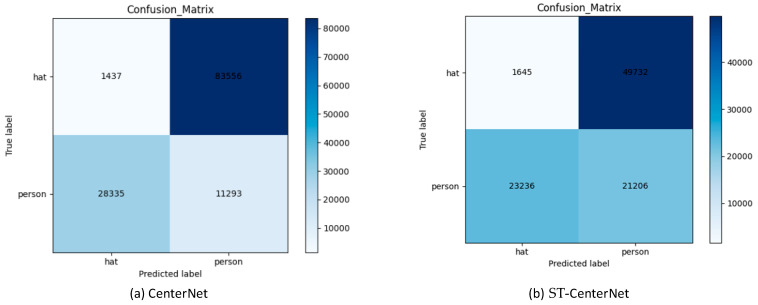
*Confusion matrix of CenterNet and ST-CenterNet technique under 200 epochs.* The desired result is an increase in the number of correctly identified hats and persons. Each figure demonstrates the prediction results based on different categories between CenterNet (**a**) and the proposed method ST-CenterNet (**b**).

The confusion matrix is a situation analysis table that summarizes the prediction results of the classification model in deep learning. It summarizes the records in the data set in the form of a matrix according to the true category and the category judgment standard predicted by the classification model. The confusion matrix generated by the CenterNet and ST-CenterNet technique on the classification of hat and person under 200 epochs is in Figure 9. The figure (a) shows that the CenterNet model has categorized 1437 targets into hat, 11,293 targets into person. The figure (b) shows that the ST-CenterNet model has categorized 1645 targets into hat, 21,206 targets into person. It can be found that the proposed ST-CenterNet model effectively increases the number of correct recognitions of hat and person.

**Figure 10:**
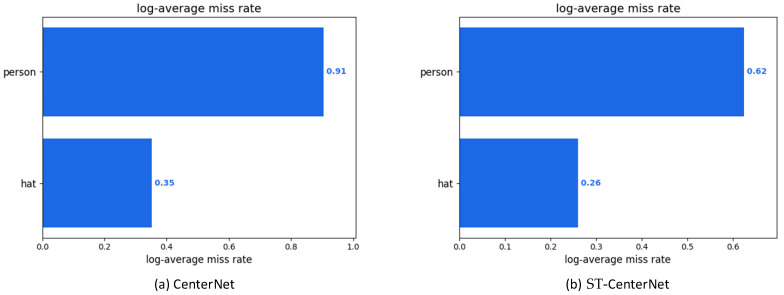
*MR^−2^ of CenterNet and our method ST-CenterNet on the SHWD.* The desired result is a reduction in the value of MR^−2^ in each target category. Figures show MR^−2^ values of different categories. Each figure demonstrates the miss rate values based on different categories between CenterNet (**a**) and our method ST-CenterNet (**b**).

In addition, we adopt the log-average miss rate (MR^−2^) to evaluate the performance of the proposed detector. The purpose is mainly to verify the effectiveness of the proposed model in reducing the proportion of missed detection of the detection results. MR^−2^ represents the miss rate of the model, and the pedestrian detector performance is measured by the curve of the average miss rate versus False Positives Per Image (FPPI) as the abscissa, and Log (MR) as the ordinate. The expected result is a reduction of MR^−2^. As can be seen in Figure 10, the proposed method shows a reduction in MR^−2^ for both types of targets detected (hat and person), when compared to the original model.

## 7. Discussion

The experimental results on SHWD show that the mAP index of the model is improved by 13.9% after replicating and flipping small targets through the SSTRA. Besides, the TAFEM considerably improves the ability of feature extraction, and the mAP of the model is increased by 13.22% compared with the original model. Compared with the CenterNet, the mAP index of the proposed algorithm is increased by 18.08%, which proves the superiority of ST-CenterNet in small target detection. SOTA performance on various methods demonstrates the superiority of ST-CenterNet in small target detection. SSTRA and AFPN can be combined with diverse detectors and various backbones to strengthen small target detection. This ability is transferrable to more specific situations. During the experiment, we found that there are still some missing targets that have not been detected, so the future improvement needs to further strengthen the capture of shallow feature information. We may consider trying to add learning to strengthen the correlation between parts using a self-attention mechanism.

## 8. Conclusions

Our proposed algorithm for small target detection with adaptive data enhancement aims to solve the lack of shallow feature information extraction and insufficient numbers of small targets. SSTRA utilizes a screening and oversampling method, which brings great benefits to increasing the number of samples and to detection effectiveness, while maintaining the total quantity of images in the dataset. TAFEM adopts the combination of ResNet-50 and AFPN to enhance the performance of extracting shallow semantic information, so as to obtain more complete semantic feature information. For future work, we are committed to using a more lightweight network to reduce the computational complexity of the model parameters. We intend to study the combination with insights from the recently proposed transformer model to accurately deepen the self-attention mechanism and more perfectly explore the relationship between different levels of feature maps.

## Figures and Tables

**Table 1:** Summary of related improvement methods.

Improvement	Literature	Backbone	Method	Merits	Limitations
Data Enhancement	Mishra [38]	ImageNet	Object-aware cropping.	Increases the sampling rate.	Poor samples may be selected, increasing the prediction error.
	Zhang [39]	ResNet	Mix-up.	Has good robustness to data with noisy labels and adversarial sample attacks.	May crop blocks to nonimportant regions or occlude important regions.
	Chen [40]	ResNet	Scale transform.	Information fed back through the optimization process dynamically guides data preparation.	The uneven distribution of objects at different scales will greatly affect the detection quality.
Feature Enhancement	Liu [33,34]	VGG16	Multi-scale feature pyramid.	The multi-scale feature pyramid is used to detect objects of different scales and aspect ratios.	Poor detection effect for small targets.
	Belfodil [42]	VGG16	Lightweight feature fusion.	Fast speed; merges feature maps of different layers.	Having duplicate boxes; shallow feature maps have insufficient representation ability.
	Zhang [43]	ResNet	A “wide-narrow-wide” structure.	Has a large improvement compared to SSD for detection accuracy.	Slow speed.
Attention Mechanism	Dai [53]	ResNet	Multi-scale channel attention.	Recognizes and detects objects under extreme scale changes.	Limited scenario; Not necessarily adaptively fusing the received features.
	Yu [55]	DenseNet	Dense connected convolutional networks.	Enhance the semantic information of small targets in shallow features.	Addition combined with identity functions hinder information propagation.
	Liu [56]	Deeplab-VGG16	Dilated convolution; add inception structures.	Considers the relationship between the size and eccentricity of the receptive field.	Low speed and expensive computation.
Angle Classfication	Han [57]	RetinaNet	Replacing the dire-ctional bounding box by the regression output.	The aligned features are more beneficial for learning rotated targets.	Requires heuristics to define anchors and complex RoI operations.
	Yang [58]	RetinaNet	Replacing the sparse coding labels with dense coding labels.	Eliminates periodicity of angle and adjust adaptively according to the aspect ratio.	Needs a longer number of bits for encoding.
	Yang [59]	RetinaNet	Using the Gaussian Wasserstein distance to describe the rotated boxes distance.	Avoids the rotation angle regression interval discontinuity and the square problem.	Needs a longer number of bits for encoding, and the output is heavy.

**Table 2:** Experimental environment configuration.

Name	Configuration
CPU	Xeon E5-2690 V4 @2.20 GHz
Memory	11GB
GPU	NVIDIA GeForce GTX 2080Ti
GPU Accelerated Libraries	CUDA10.1.105, cuDNN7.6.5
Deep Learning Framework	Python3.7.2, Pytorch1.4.0
Operating System	Ubuntu20.04

**Table 3:** Comparison of different algorithm configurations.

Algorithm	Backbone	Input Size	Precision	Recall	F1-Score	mAP	FPS
SSD [33,34]	VGG16	512 × 512	92.09	52.55	0.65	73.77	23.39
YOLOv3 [64]	DarkNet53	512 × 512	85.05	53.31	0.66	69.17	32.54
YOLOv3_EfficientNet [65]	EfficientNet	512 × 512	88.34	29.25	0.44	61.79	20.57
YOLOv4 [66]	CSPDarkNet53	512 × 512	49.84	40.74	0.45	40.84	7.39
YOLOv4_ResNet	ResNet50 [25]	512 × 512	68.44	58.00	0.63	59.48	12.83
YOLOv5	CSPDarkNet53	512 × 512	92.54	66.93	0.78	78.57	9.35
YOLOX [37]	CSPDarkNet53	512 × 512	92.21	78.10	0.85	86.09	15.10
CenterNet [23]	ResNet50	512 × 512	88.90	56.12	0.69	70.98	32.78
ST-CenterNet	ResNet50	512 × 512	94.19	71.88	0.82	89.06	28.69

**Table 4:** Effect on component in ST-CenterNet.

Method	Precision	Recall	F1-Score	mAP	FPS
CenterNet [23]	88.90	56.12	0.69	70.98	32.78
CenterNet+SSTRA	94.08	75.15	0.84	84.88	15.25
CenterNet+TAFEM	94.56	72.90	0.82	84.20	16.64
ST-CenterNet	94.19	71.88	0.82	89.06	28.69

## Data Availability

The datasets analyzed during the current study are available in the Github repository, GitHub—tanypadam/Safety-Helmet-Wearing-Dataset: Safety helmet wearing detect dataset, with pretrained model.
